# Data-Driven Prediction of Induced Voltage in CT-Based Magnetic Energy Harvesting Systems Considering Nonlinear B–H Characteristics

**DOI:** 10.3390/ma19143002

**Published:** 2026-07-12

**Authors:** Seunggyun Byeon, Minjoong Kim, Jihwan Song

**Affiliations:** 1 Department of Mechanical Engineering, Sogang University, 35 Baekbeom-ro, Mapo-gu, Seoul 04107, Republic of Korea; sgbyeon@sogang.ac.kr; 2 Department of Mechanical Engineering, Hanbat National University, 125 Dongseodae-ro, Yuseong-gu, Daejeon 34158, Republic of Korea; kimminjoong.mmm@gmail.com

**Keywords:** current transformer, magnetic energy harvesting, nonlinear B–H curve, deep neural network, surrogate model, data-driven modeling

## Abstract

This paper presents a study on the development of a deep neural network (DNN)-based surrogate model for rapid induced voltage prediction in current transformer (CT)-based magnetic energy harvesting systems. CT-based magnetic energy harvesters are promising self-powered energy sources for low-power electronic devices, but their output performance is strongly affected by the nonlinear magnetic behavior of the core material. Therefore, the accurate prediction of the induced voltage is important for device design. However, evaluating the voltage response under various magnetic material characteristics and operating conditions through repeated electromagnetic simulations requires considerable computational effort. In this study, nonlinear B–H curves were parameterized using an arctangent-based model, and electromagnetic simulations were performed by varying the magnetic material parameters, primary current, and load resistance. The resulting dataset was used to train and validate the DNN surrogate model. The trained model showed high prediction accuracy, with an *R*^2^ value greater than 0.99 and low prediction errors. It also reproduced the RMS induced voltage trends for different magnetic material characteristics and operating conditions and was further used for maximum power point analysis within the investigated parameter range. These results indicate that the proposed surrogate model can reduce the need for repeated electromagnetic simulations and support the efficient design exploration of CT-based magnetic energy harvesting systems.

## 1. Introduction

Current transformer (CT)-based magnetic energy harvesting (MEH) technology has attracted considerable attention as a self-powered energy supply method for power infrastructure environments, and related studies have been continuously reported [1,2,3]. MEH systems can be utilized as power sources for condition-monitoring devices by harvesting alternating magnetic fields in environments where external power supply or battery replacement is difficult, such as transmission and distribution lines [4,5]. In addition, compared with other energy harvesting technologies, including vibration-, wind-, and solar-based approaches [6,7,8,9,10,11,12], MEH has been regarded as a promising energy source for power line applications because of its lower dependence on environmental conditions and higher power density [13].

For the practical application of CT-based MEH systems as power sources, sufficient output power should be secured to operate condition-monitoring devices even under varying operating conditions such as primary current and load resistance [14]. Therefore, during the design stage of CT-based MEH systems, it is necessary to evaluate the output power according to design parameters, including magnetic material properties and operating conditions, in order to identify the design space satisfying the required output performance.

To evaluate the output power during the design process, theoretical approaches based on electromagnetic induction principles and circuit-based models have been employed to calculate the magnetization behavior of the magnetic core under operating conditions and subsequently estimate the output characteristics [15,16,17]. When the B–H characteristics and geometry of the CT core change, the saturation behavior and effective permeability of the core also vary, and these properties are difficult to estimate consistently for different materials and geometries [18]. Consequently, theoretical approaches that rely on simplified magnetic properties have limitations in systematically investigating output characteristics across a broad design space including variations in material properties and geometry.

To overcome the limitations of theoretical analysis, experimental performance evaluations based on the direct implementation and measurement of CT-based MEH systems have also been conducted [19,20,21,22]. Although experimental measurements provide reliable output power evaluation under actual operating conditions, repeated core fabrication and measurement processes are required to identify design parameters satisfying the target output performance. As a result, similarly to theoretical approaches, it is difficult to systematically explore a wide design space, and experimental-only approaches involve significant time and cost constraints in deriving feasible design conditions.

Meanwhile, electromagnetic simulations have been widely utilized as an alternative approach to compensate for the limitations associated with magnetic material modeling and repeated fabrication in previous methods [23,24,25]. Electromagnetic simulations can accurately calculate output power by considering nonlinear B–H characteristics and operating conditions. However, nonlinear electromagnetic field analysis requires significant computational resources even for a single simulation. Furthermore, repeated simulations over multiple design parameters are necessary to identify feasible design conditions, resulting in a considerable computational cost during the design and evaluation process.

Therefore, a rapid evaluation method capable of reducing the computational cost of repeated electromagnetic simulations is required for the design of CT-based MEH systems. Such an approach would enable the rapid comparison of output characteristics under various design conditions and facilitate the identification of feasible design spaces for high-performance CT core design.

Recently, data-driven surrogate models have emerged as an effective approach for replacing computationally expensive numerical simulations in various engineering design problems. By learning the relationship between design variables and system responses from simulation data, surrogate models can provide rapid predictions while maintaining reasonable accuracy [26,27,28,29].

Herein, a deep neural network (DNN)-based surrogate model is proposed to predict the induced voltage for evaluating the output power of CT-based MEH systems. The dataset is constructed using electromagnetic simulations, and the model predicts the induced voltage based on two parameters that define the nonlinear B–H curve, the primary current and load resistance. The model is designed to provide predictions consistent with simulation results, even for combinations of B–H curve parameters and operating conditions not included in the training dataset. Through this approach, induced voltage responses can be rapidly evaluated across diverse magnetic material properties and operating conditions without repeated electromagnetic simulations. This enables efficient design space exploration and provides a practical framework for the future optimization of CT-based MEH systems. Table 1 summarizes the previously reported approaches for output performance evaluation in CT-based MEH systems and compares them with the present surrogate modeling approach. To the best of our knowledge, surrogate modeling for direct induced voltage prediction considering both nonlinear magnetic material characteristics and operating conditions has not been reported for CT-based MEH systems.

## 2. Methodology

**Figure 1:**
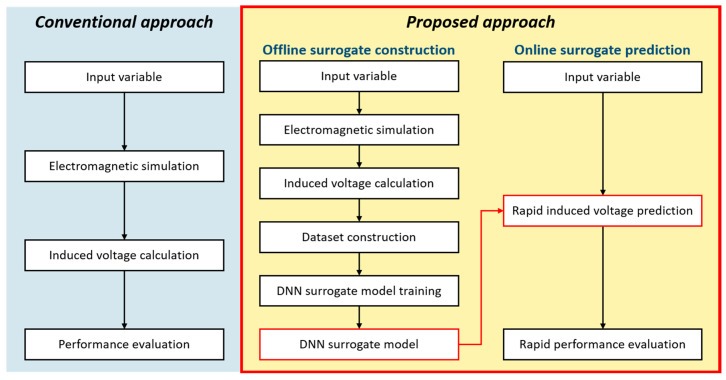
Overall workflow of proposed DNN-based surrogate modeling framework. Unlike the conventional approach requiring repeated electromagnetic simulations, the proposed approach constructs a DNN surrogate model offline and uses it for rapid online induced-voltage prediction and performance evaluation.

This section describes the methodology for developing the DNN-based surrogate model for induced voltage prediction in the CT-based MEH system. The workflow is shown in Figure 1. Electromagnetic simulations were performed to generate induced voltage data by varying nonlinear magnetic material properties and operating conditions. The nonlinear magnetic behavior of the core material was represented using an arctangent-based B–H curve model, and the resulting simulation data were organized into input and output variables for model training. A multilayer perceptron-based DNN model was then trained to learn the nonlinear relationship between the B–H curve parameters, operating conditions, and induced voltage. Once trained, the surrogate model enables direct induced voltage prediction without repeated electromagnetic simulations during performance evaluation.

### 2.1. CT-Based MEH System Configuration

**Figure 2:**
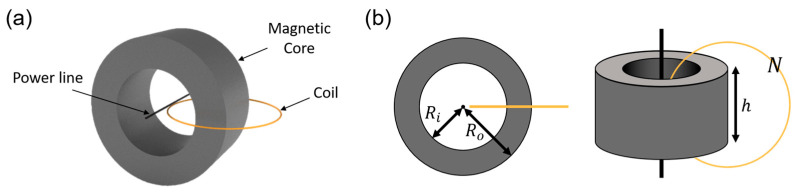
Schematic configuration of CT-based MEH system: (**a**) three-dimensional model consisting of magnetic core, power line, and coil; (**b**) geometric parameters of toroidal core and coil. Outer radius (*R_o_*), inner radius (*R_i_*), and height (*h*) were set to 62.5 mm, 37.5 mm, and 60 mm, respectively.

An electromagnetic simulation model was constructed using ANSYS Maxwell 3D 2021 R2 (Ansys, Inc., Canonsburg, PA, USA) to analyze the electromagnetic behavior of the CT-based MEH system and calculate the induced voltage. Figure 2 shows the schematic configuration of the CT-based MEH system. The model consists of a toroidal magnetic core, a power line, and a coil. Detailed coil geometry and placement relative to the toroidal core and power line, including the coil dimensions and number of turns, are shown in Figure S1. When an alternating current flows through the power line, a time-varying magnetic field is generated around the conductor, producing a time-varying magnetic flux in the core. This variation in magnetic flux induces a voltage in the coil according to electromagnetic induction. The electromagnetic simulation was performed using finite element analysis based on Maxwell’s equations, and Equations (1)–(4) represent the governing equations used for the electromagnetic field analysis.

(1) $$\begin{array}{c} \nabla \times H=J+\frac{\partial D}{\partial t}\; \end{array}$$

(2) $$\begin{array}{c} \nabla \cdot B=0\; \end{array}$$

(3) $$\begin{array}{c} \nabla \times E=-\frac{\partial B}{\partial t}\; \end{array}$$

(4) $$\begin{array}{c} \nabla \cdot D=\rho \; \end{array}$$

These governing equations were used to calculate the magnetic field generated by the alternating current in the power line and the magnetic flux distribution inside the core. The induced voltage in the coil was then obtained from the time variation in the magnetic flux. The induced voltage was selected as the primary response variable for evaluating the output characteristics of the CT-based MEH system. In addition, the nonlinear magnetic behavior of the core material was incorporated into the electromagnetic simulation by assigning the B–H curve to the magnetic core.

### 2.2. Arctangent-Based B–H Curve Modeling for Electromagnetic Simulation

The B–H curve defines the relationship between the magnetic flux density *B* and the magnetic field intensity *H* and is used to characterize the nonlinear magnetic properties of the core material. An arctangent-based B–H curve model, expressed in Equation (5), was employed to represent the magnetic saturation behavior using a limited number of parameters.

(5) $$\begin{array}{c} B\left(H\right)=a\cdot \tan^{-1}\left(\frac{H}{b}\right)+\mu_{0}\cdot H\; \end{array}$$

**Figure 3:**
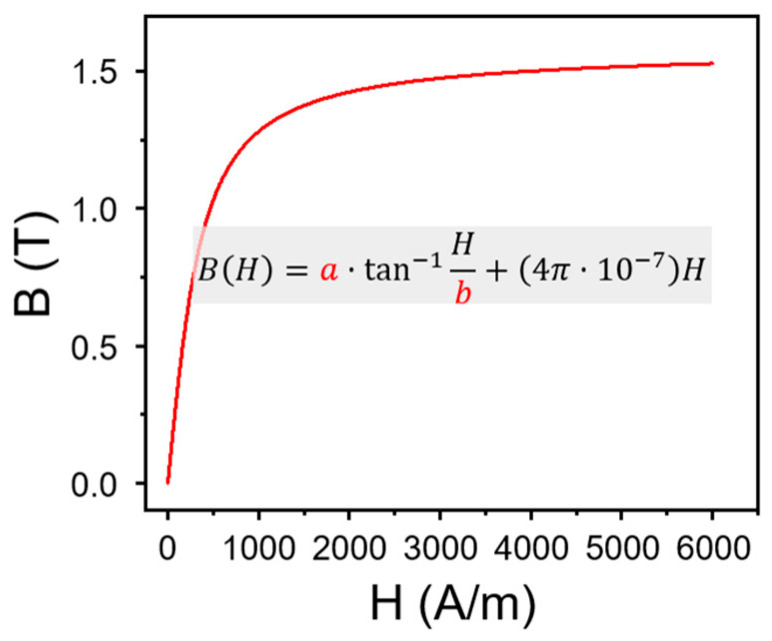
Arctangent-based representation of B–H curve.

Here, *a* and *b* are the parameters of the arctangent-based B–H curve model, and *μ*_0_ is the vacuum permeability (*μ*_0_ = 4π × 10^−7^ H/m), which represents the permeability of free space. Figure 3 shows a representative arctangent-based B–H curve expressed by Equation (5).

The arctangent-based model can continuously describe both the initial permeability and saturation regions using a limited number of parameters and can represent different nonlinear magnetic characteristics by varying the parameter values [30]. In addition, this model has been reported as a technical model for representing B–H saturation magnetization curves in SPICE, FEM, and MoM simulations, with reported fitting error parameter values of 3.1–14% and *R*^2^ values of 0.97–0.996 for representative soft magnetic materials, including Mn–Zn ferrite, amorphous alloy, and Finemet-type nanocrystalline material [31]. Therefore, the arctangent-based B–H model was adopted as a compact parameterization method to efficiently and systematically represent nonlinear magnetic saturation behavior.

Parameter *a* controls the saturation level of the magnetic flux density, whereas parameter *b* controls the low-field slope characteristic and saturation knee behavior of the B–H curve. In CT-based MEH systems, obtaining sufficient magnetic flux under limited primary current excitation while avoiding early core saturation is important for stable voltage generation. Therefore, *a* and *b* were used as parameters to represent the effective nonlinear saturation characteristics that affect the induced voltage response.

The selected ranges of *a* and *b* were not intended to cover the complete magnetic behavior of all possible CT core materials but were defined as the training domain for the nonlinear B–H saturation characteristics considered in the target CT-based MEH application [4]. Accordingly, these ranges should be interpreted as the validity domain of the trained surrogate model.

In addition, the arctangent-based B–H model was used as an anhysteretic B–H representation to analyze the effect of nonlinear saturation characteristics on the induced voltage response in a parameterized form, rather than as a model that reproduces hysteresis behavior itself [31]. Accordingly, hysteresis loop characteristics such as remanence and coercivity are not explicitly included in this parameterization.

**Figure 4:**
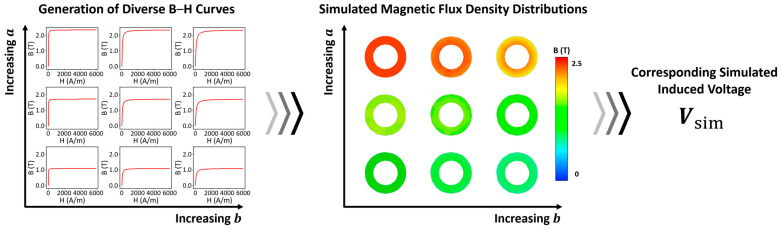
Schematic workflow of arctangent-based B–H curve parameterization and electromagnetic simulation: generation of nonlinear B–H saturation curves with varying *a* and *b*; simulated magnetic flux density distributions for corresponding B–H curves; and corresponding simulated induced voltage *V*_sim_.

Figure 4 schematically illustrates the process by which the arctangent-based B–H curve parameterization is applied to the electromagnetic simulation framework. Various nonlinear B–H saturation curves are generated from different combinations of *a* and *b*, and the generated B–H curves are applied to the electromagnetic simulation as the B–H characteristics of the magnetic core material. The variation in the B–H characteristics is reflected in the magnetic flux density distribution inside the core, and the corresponding induced voltage *V*_sim_ is calculated for each simulation case. Through this procedure, an electromagnetic simulation framework was established in which the nonlinear saturation characteristics of the core material are reflected in the induced voltage response.

### 2.3. Simulation-Based Dataset Construction

A dataset was constructed to train the DNN-based surrogate model for induced voltage prediction in the CT-based MEH system. The input variables consist of the B–H curve parameters *a* and *b*, the primary current *I*_p_ flowing through the power line, and the load resistance *R*_L_, while the output variable is the induced voltage *V* generated in the coil. The primary current *I*_p_ and induced voltage *V* were expressed as RMS values throughout dataset construction, model training, and performance evaluation. The B–H curve parameters represent the nonlinear magnetic characteristics of the core material, whereas the primary current and load resistance define the operating conditions of the CT-based MEH system. The output power used to evaluate the energy harvesting performance is given by Equation (6).

(6) $$\begin{array}{c} P_{\mathrm{out}}=\frac{V_{\mathrm{rms}}^{2}}{R_{L}} \end{array}$$

where *P*_out_ denotes the output power, *V*_rms_ denotes the RMS induced voltage, and *R*_L_ denotes the load resistance. Induced voltage was selected as the prediction target because it is the primary electromagnetic response directly obtained from the electromagnetic simulation, while output power can be subsequently calculated from the predicted voltage and load resistance. Accordingly, the relationship between the selected input variables and the prediction target is expressed in Equation (7).

(7) $$\begin{array}{c} {\hat{V}}_{i}=f_{\theta }\left(a,b,I_{p},R_{L}\right)\; \end{array}$$

Here, ${\hat{V}}_{i}$ denotes the predicted induced voltage. To evaluate the generalization performance of the proposed model under various magnetic material properties and operating conditions, electromagnetic simulations were performed by varying the input variables within predefined ranges.

The dataset was constructed from the induced voltage responses calculated under each input condition and was divided into training, validation, and test datasets consisting of 1250, 300, and 750 data samples, respectively. Note that the term ‘validation’ in this study refers to the machine learning practice of model evaluation and hyperparameter selection using simulation-generated data and should not be interpreted as experimental validation against physical measurements. The test dataset was used to evaluate the generalization performance and prediction reliability of the proposed model under conditions not used for model training.

Min–Max normalization was applied to all input variables to reduce the effect of scale differences during model training. The normalization parameters were determined from the training dataset, and the same transformation was then applied to the validation and test datasets to maintain consistency across the datasets. The ranges of the input variables used for data generation are summarized in Table 2.

### 2.4. DNN Surrogate Model Development

**Figure 5:**
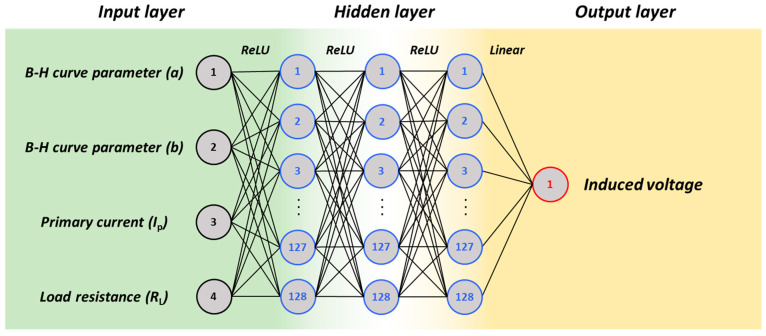
DNN-based surrogate model architecture. The black circles represent the four input variables, the blue circles represent the 128 neurons in each hidden layer, and the red circle represents the single output, induced voltage.

A DNN-based surrogate model was constructed to replace repeated electromagnetic simulations and directly predict the induced voltage from the input variables. The model uses the B–H curve parameters and operating conditions as inputs. The overall architecture is shown in Figure 5.

The DNN architecture used in this study is a conventional fully connected feedforward network, and the main contribution of this work is not the development of a new neural network structure. Rather, the proposed framework integrates the arctangent-based B–H parameters *a* and *b*, primary current *I*_p_, and load resistance *R*_L_ into a simulation-trained surrogate model to predict the induced voltage response of CT-based MEH systems. This formulation enables efficient design space exploration by accounting for both parameterized magnetic saturation characteristics and operating conditions without repeated electromagnetic simulations.

The model was based on a multilayer perceptron architecture consisting of an input layer, three hidden layers, and an output layer. Each hidden layer was fully connected and contained 128 neurons. A Rectified Linear Unit (ReLU) activation function was applied to the hidden layers to learn the nonlinear relationship between the input variables and the induced voltage, as defined in Equation (8).

(8) $$\begin{array}{c} f\left(x\right)=\max\left(0,x\right)\; \end{array}$$

Model training was performed using the Adam optimizer with mean squared error (MSE) as the loss function. Early stopping was applied to reduce overfitting and improve the generalization capability of the model. Training was terminated when the validation loss did not decrease for more than 200 consecutive epochs, and the model parameters corresponding to the minimum validation loss were selected as the final model. The main hyperparameters and training configurations are summarized in Table 3.

## 3. Results and Discussion

The electromagnetic simulation framework adopted in the present study was based on our previous CT-based MEH model, which was experimentally evaluated using a prototype system [32]. In the previous study, the electromagnetic simulation was performed using a fitted B–H curve obtained from the magnetic material data. In contrast, the present study reformulated the magnetic material representation using arctangent-based B–H parameterization to construct the simulation dataset for surrogate model training. Therefore, an additional comparison was performed to examine whether the arctangent-based B–H representation preserves the system-level power–resistance response of the previously experimentally evaluated simulation framework.

Figure S3a compares the fitted B–H curve used in the previous study with the B–H curve fitted using the arctangent function [32]. The two B–H representations exhibited similar nonlinear saturation characteristics, indicating that the arctangent-based representation reasonably approximates the main saturation behavior of the previous fitted B–H curve.

Figure S3b–f show the power–resistance characteristics obtained using the two B–H representations under different primary current conditions. The simulation results obtained using the arctangent-fitted B–H curve showed output power responses similar to those of the previous simulation results. In particular, the formation of the maximum power point with increasing load resistance and the saturation tendency in the high-resistance region were consistently reproduced. These results indicate that the nonlinear saturation characteristics of the previous B–H curve were appropriately reflected in the system-level simulation response obtained using the arctangent-based B–H representation.

In addition, the experimental results reported in the previous study were compared with the present arctangent-based simulation results to quantitatively evaluate the discrepancy between the measured and simulated power responses [32]. The corresponding error metrics are summarized in Table S1. The results show that the present arctangent-based simulation generally reproduces the power–resistance tendency of the experimental results, although a certain level of discrepancy remains. Since the proposed DNN surrogate model was shown to reproduce the arctangent-based electromagnetic simulation results with high accuracy, the discrepancy between the surrogate prediction and experimental measurements is expected to be comparable to the simulation–experiment discrepancy observed in the present comparison, within the scope of the adopted B–H representation and operating conditions. The remaining discrepancy may originate from simplifications in the arctangent-based B–H representation, uncertainties in magnetic material properties, manufacturing tolerances, and differences in experimental measurement conditions. Therefore, incorporating experimentally characterized magnetic properties into the electromagnetic simulation model and surrogate modeling framework could further improve the practical predictive capability under operating conditions.

After examining the consistency of the arctangent-based simulation framework with the previous experimental results, the DNN-based surrogate model was evaluated to determine how accurately it reproduced the induced voltage responses obtained from electromagnetic simulations. The performance of the surrogate model was analyzed from three aspects: training convergence, prediction trends for selected test cases, and quantitative prediction accuracy on the test dataset.

**Figure 6:**
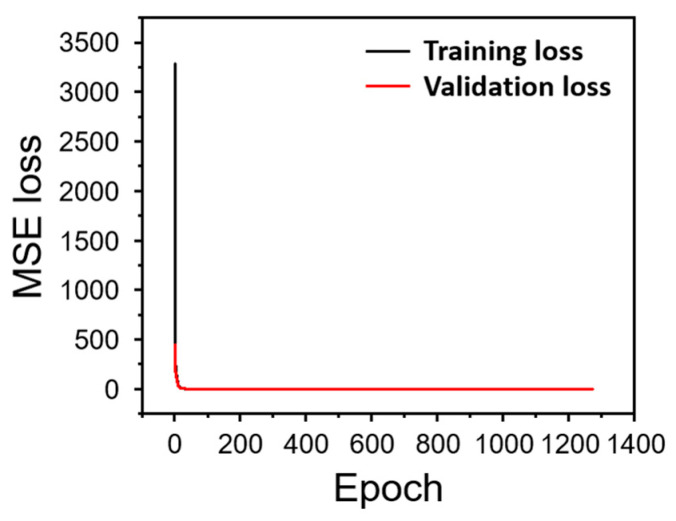
Training and validation loss of proposed DNN surrogate model.

Figure 6 shows the training and validation loss curves of the DNN model. Both losses decreased rapidly during the early stage of training and then converged stably, while the gap between them did not increase noticeably. The final model was selected based on the minimum validation loss during early stopping. The final training and validation losses were 0.2471 and 0.3809, respectively, suggesting that the model was trained stably without apparent overfitting.

**Figure 7:**
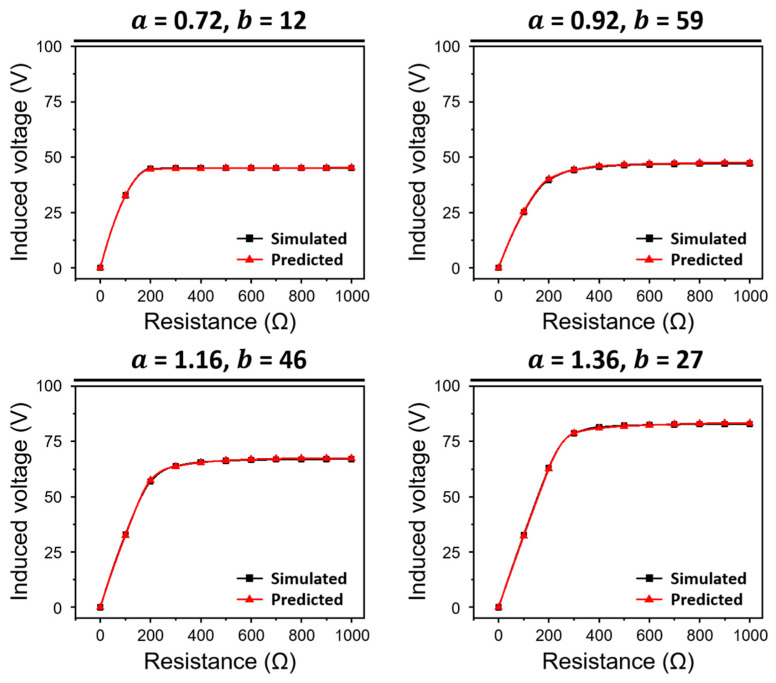
Comparison between simulated and predicted RMS induced voltage–resistance characteristics for test cases with different B–H curve parameter conditions.

**Figure 8:**
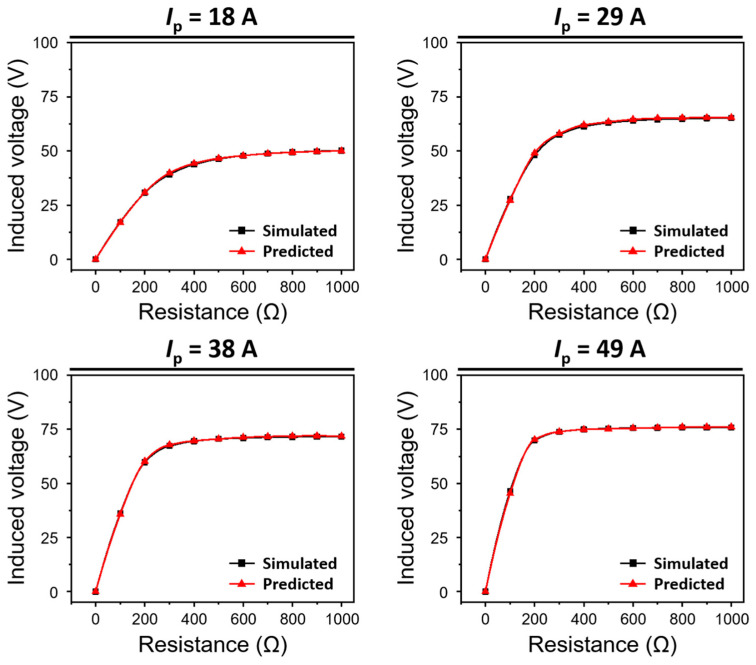
Comparison between simulated and predicted RMS induced voltage–resistance characteristics for test cases with different primary current conditions.

Figure 7 compares the induced voltage predictions with the electromagnetic simulation results for test cases with different combinations of B–H curve parameters. The DNN model reproduced the saturation behavior of the induced voltage with increasing resistance, showing close agreement with the simulation results even under different magnetic saturation characteristics. This comparison demonstrates that the model can capture the effect of nonlinear magnetic material properties on the induced voltage response. Figure 8 presents the induced voltage prediction results for test cases with different primary current conditions. The changes in voltage magnitude and saturation behavior according to the primary current were also predicted in close agreement with the simulation results, demonstrating stable prediction performance under varying operating conditions.

Although the simulation and DNN-predicted curves are closely overlapped in Figure 7 and Figure 8, this agreement does not indicate perfect prediction. The prediction error was quantitatively evaluated using the independent test dataset, as summarized in Table 4. The high prediction accuracy is attributed to the use of noise-free electromagnetic simulation data and the continuous variation in the induced voltage response within the investigated parameter space.

**Figure 9:**
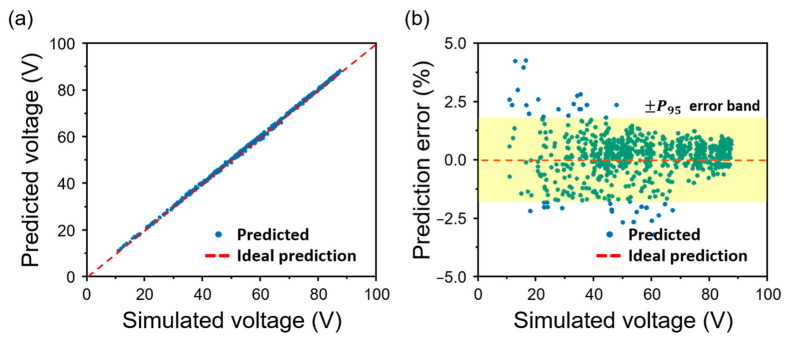
Prediction results and error distribution of proposed DNN surrogate model: (**a**) comparison between simulated and predicted RMS induced voltages for test dataset; (**b**) percent prediction error as function of simulated voltage, with ±*P*_95_ error band corresponding to ±1.323%.

Figure 9 summarizes the prediction results and error distribution for the test dataset. As shown in Figure 9a, the predicted induced voltages were distributed near the ideal prediction line, *y = x*, indicating high agreement between the DNN predictions and electromagnetic simulation results. The percent prediction error distribution in Figure 9b also shows that most prediction errors were confined within a narrow range. The percent prediction error was calculated using Equation (9), and the *P*_95_ metric was calculated as the 95th percentile of the absolute percent prediction error.

(9) $$\begin{array}{c} P_{95}={\mathrm{Percentile}}_{95}\left(\;\left|\frac{V_{i}-{\hat{V}}_{i}}{V_{i}}\right|\times 100\right)\; \end{array}$$

Here, $V_{i}$ and ${\hat{V}}_{i}$ denote the simulated and predicted induced voltages, respectively. The prediction accuracy of the proposed model was quantitatively evaluated using the mean squared error (MSE), root mean squared error (RMSE), mean absolute error (MAE), and coefficient of determination (*R*^2^), as defined in Equations (10)–(13).

(10) $$\begin{array}{c} \mathrm{MSE}=\frac{1}{N}\overset{N}{\underset{i=1}{\sum }}{\left(\;V_{i}-{\hat{V}}_{i}\right)}^{2}\; \end{array}$$

(11) $$\begin{array}{c} \mathrm{RMSE}=\sqrt{\frac{1}{N}\overset{N}{\underset{i=1}{\sum }}{\left(\;V_{i}-{\hat{V}}_{i}\right)}^{2}}\; \end{array}$$

(12) $$\begin{array}{c} \mathrm{MAE}=\frac{1}{N}\overset{N}{\underset{i=1}{\sum }}\left|\;V_{i}-{\hat{V}}_{i}\;\right|\; \end{array}$$

(13) $$\begin{array}{c} R^{2}=1-\frac{\sum_{i=1}^{N}{\left(V_{i}-{\hat{V}}_{i}\right)}^{2}}{\sum_{i=1}^{N}{\left(V_{i}-\bar{V}\right)}^{2}}\; \end{array}$$

**Figure 10:**
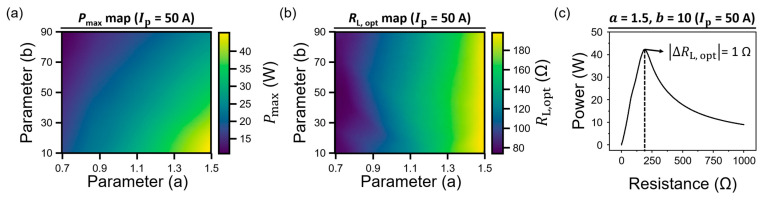
Maximum power point analysis using trained DNN surrogate model: (**a**) *P*_max_ map; (**b**) *R*_L,opt_ map; (**c**) load resistance optimization result for selected condition based on output power response.

The trained DNN surrogate model was used to obtain the *P*_max_ and *R*_L,opt_ maps in the *a*–*b* parameter space. For each *a*–*b* condition, the output power was calculated by substituting the induced voltage predicted by the surrogate model into Equation (6), and *R*_L_ was varied at 1 Ω intervals to extract *P*_max_ and the corresponding *R*_L,opt_. Figure 10a,b show the *P*_max_ map and *R*_L,opt_ map obtained at *I*_p_ = 50 A, respectively. Within the investigated parameter range, *P*_max_ tended to increase as *a* increased and *b* decreased, and the highest *P*_max_ was obtained at *a* = 1.5 and *b* = 10. This indicates that an increased saturation-related flux density level and a sharper magnetization transition in the low-field region can increase the magnetic flux variation in the core, thereby contributing to enhanced output power. Figure 10c compares the power–resistance response for *a* = 1.5, *b* = 10, and *I*_p_ = 50 A. Under this condition, the reference *R*_L,opt_ identified from the electromagnetic simulation was 190 Ω, whereas the *R*_L,opt_ extracted using the surrogate model was 191 Ω, showing good agreement with the simulation-based reference value.

To examine the change in the *P*_max_ distribution with primary current, *P*_max_ maps were generated at *I*_p_ = 10, 30, and 50 A using the same procedure. As shown in Figure S2, the highest *P*_max_ was obtained at *a* = 1.5 and *b* = 10 for all examined primary current conditions. However, although the condition yielding the highest *P*_max_ was the same across the examined current range, the relative influence of *a* and *b* on *P*_max_ varied with the primary current level. At lower *I*_p_, the magnetic response is more strongly associated with the low-field region of the B–H curve; therefore, the influence of *b*, which determines the initial slope and transition behavior of the B–H curve, becomes more pronounced. In contrast, as *I*_p_ increased, the magnetic response extends toward a higher-flux-density region, where the saturation-related flux density level governed by *a* becomes increasingly important. Therefore, within the investigated range, a higher saturation-related flux density level combined with a sharper transition characteristic was the most favorable for improving *P*_max_, while the relative importance of *a* and *b* depended on the target operating current range.

The quantitative prediction performance metrics and baseline comparison results are summarized in Table 4. To evaluate prediction performance relative to representative baseline regression models, GPR [33], RF [34], and the MLP-based DNN [35] were compared using the same training, validation, and test datasets and the same evaluation metrics. The MLP-based DNN achieved the lowest MSE, RMSE, and MAE and the highest *R*^2^ among the compared models. GPR also showed good prediction performance but exhibited higher prediction errors than the MLP-based DNN. RF can also be used as a regression baseline model. However, it showed larger prediction errors than both GPR and the MLP-based DNN in the present comparison. Therefore, the MLP-based DNN provided the most accurate deterministic surrogate for reproducing the electromagnetic simulation results within the investigated parameter ranges. In addition to prediction accuracy, the trained surrogate model substantially reduced the computational time required for performance evaluation. For fixed B–H curve parameters and a fixed primary current, an electromagnetic simulation required 432 s to evaluate the induced voltage response over the load resistance range, whereas the trained DNN model completed the same prediction task in approximately 3 s. This reduction in evaluation time demonstrates the computational advantage of the proposed surrogate model for iterative design space exploration and design evaluation.

## 4. Conclusions

In this study, a DNN-based surrogate model was proposed to directly predict the induced voltage of a CT-based MEH system using electromagnetic simulation data. The model was specifically designed to capture the influence of the nonlinear magnetic properties of the CT core material and operating conditions on the induced voltage response.

To evaluate the proposed approach, an electromagnetic simulation model was developed using ANSYS Maxwell 3D, where the saturation behavior of the magnetic material was represented using an arctangent-based B–H curve model. Based on the electromagnetic simulation framework, a dataset of induced voltages was generated for various combinations of magnetic material properties and operating conditions. A DNN was then developed to learn the nonlinear relationship between the input variables and the induced voltage response. A comparison with the simulation results demonstrated that the proposed model accurately reproduced both the magnitude and saturation behavior of the induced voltage for test cases with different B–H curve parameters and operating conditions. In particular, strong agreement with the simulation results and low error metrics were obtained for the test dataset, indicating robust generalization performance within the investigated B–H parameter ranges and operating conditions.

Furthermore, the surrogate-based analysis was extended from induced voltage prediction to output power characterization by using the predicted RMS induced voltage together with load resistance. Through load resistance sweeps performed with the trained surrogate model, maximum power point conditions were identified within the investigated magnetic material parameter space and operating conditions. Accordingly, it was demonstrated that the proposed surrogate model can be used not only for rapid induced voltage prediction but also for design space exploration and maximum power point analysis in CT-based MEH systems.

Although an initial computational investment is required for simulation data generation and model training, these processes are performed only once. Once trained, the rapid prediction of induced voltage responses can be performed using the trained model without the need for repeated electromagnetic simulations. Therefore, the proposed DNN-based surrogate model can be used as an efficient alternative for reducing the computational burden associated with iterative analyses and repeated design evaluations in the design of CT-based MEH systems. Unlike conventional simulation-based approaches, the direct prediction of induced voltage responses can be achieved using the proposed method for nonlinear problems involving multiple coupled design variables, such as magnetic material properties, primary current, and load resistance.

The applicability of the proposed surrogate model should be interpreted within the arctangent-based B–H parameterization and the selected training domain of *a* and *b*. Although nonlinear saturation behavior can be compactly represented by the arctangent-based B–H model, the fitting accuracy of this representation may vary depending on the material-specific shape of the measured B–H curve. Therefore, if the magnetic characteristics of a target material lie outside the present parameter domain or are not sufficiently represented by the arctangent form, additional material characterization or an expanded simulation dataset may be required to ensure reliable prediction.

Although the present study focused on CT-based MEH systems, the proposed surrogate modeling strategy could be extended to other electromagnetic induction-based systems that require repeated electromagnetic simulations, such as transformers, inductors, and electromagnetic energy harvesting devices. For such extensions, the input variables and simulation dataset should be redefined according to the material properties, geometry, and operating conditions of the target system.

In future work, experimentally characterized magnetic properties will be incorporated into the electromagnetic simulation model and surrogate modeling framework, and surrogate model predictions will be directly validated using measured induced voltage responses under practical operating conditions. Beyond induced voltage prediction, this approach could also be extended to efficiency prediction by incorporating input-power- or loss-related quantities into an expanded electromagnetic simulation dataset. The extended surrogate model could then be integrated with design optimization techniques to provide a systematic and computationally efficient framework for the design space exploration of and performance enhancement in electromagnetic induction systems.

## Figures and Tables

**Table 1:** Comparison of previously reported approaches for output performance evaluation in CT-based MEH systems.

Category	Related Studies	Advantages	Limitations/Remaining Issues
Theoretical and circuit-based approaches	[15,16,17,18]	Provide physical insight and relatively fast estimation of output characteristics.	Often rely on simplified magnetic properties and assumptions; limited for systematic evaluation of nonlinear B–H effects across broad design spaces.
Experimental evaluation-based approaches	[19,20,21,22]	Provide reliable output performance evaluation under actual operating conditions.	Require repeated fabrication and measurement, leading to time and cost constraints for design space exploration.
Electromagnetic simulation-based approaches	[23,24,25]	Can consider nonlinear B–H characteristics, geometry, and operating conditions in detail.	Repeated nonlinear electromagnetic simulations impose a considerable computational cost.
Data-driven surrogate modeling approaches in related engineering design problems	[26,27,28,29]	Enable rapid prediction after learning input–response relationships from simulation or numerical data.	Have not been directly developed for CT-based MEH induced voltage prediction considering nonlinear B–H characteristics and operating conditions.
Present study	This work	Predicts induced voltage using arctangent-parameterized B–H characteristics, primary current, and load resistance.	Valid within the selected B–H parameterization and trained parameter range.

**Table 2:** Range of parameters used for dataset construction.

Parameter	Range
*a* (T)	0.7–1.5
*b* (A/m)	10–90
*I*_p_ (A)	10–50
*R*_L_ (Ω)	100–1000

**Table 3:** Hyperparameters and training configuration of proposed DNN model.

Parameter	Value
Number of hidden layers	3
Neurons per hidden layer	128
Hidden layer activation function	ReLU
Output layer activation function	Linear
Optimizer	Adam
Loss function	MSE
Learning rate	0.001
Batch size	16
Maximum epochs	3000

**Table 4:** Prediction performance comparison of GPR, RF, and proposed DNN model on test dataset.

Model	MSE (V^2^)	RMSE (V)	MAE (V)	*R* ^2^
GPR	1.3638	1.1678	0.9010	0.9961
RF	19.8941	4.4603	3.6311	0.9429
DNN	0.0969	0.3113	0.2332	0.9997

## Data Availability

The original contributions presented in this study are included in the article/Supplementary Materials. Further inquiries can be directed to the corresponding author.

## Supplementary Materials

The following supporting information can be downloaded at https://www.mdpi.com/article/10.3390/ma19143002/s1, Figure S1: Coil geometry and placement in the CT-based MEH electromagnetic simulation model: (a) coil placement around the toroidal core and enlarged coil geometry; (b) a schematic representation of the coil position relative to the core and power line, together with the coil geometric parameters and number of turns used in the simulation; Figure S2: Comparison of *P*_max_ maps in the *a*–*b* parameter space under different primary current conditions: (a) *I*_p_ = 10 A, (b) *I*_p_ = 30 A, and (c) *I*_p_ = 50 A, respectively; Figure S3: Arctangent-based B–H curve fitting and power–resistance comparison: (a) trendline-fitted and arctangent-fitted B–H curves; (b–f) simulated output power as a function of load resistance under primary current conditions of (b) *I*_p_ = 10 A, (c) *I*_p_ = 20 A, (d) *I*_p_ = 30 A, (e) *I*_p_ = 40 A, and (f) *I*_p_ = 50 A; Table S1: Error metrics of arctangent-based simulation results relative to experimental measurements.
