# Supplementary material for: Data-Driven Prediction of Induced Voltage in CT-Based Magnetic Energy Harvesting Systems Considering Nonlinear B–H Characteristics
Source: Materials (Basel). 2026 Jul 12;19(14):3002. doi: 10.3390/ma19143002 (PMC13412951; doi:10.3390/ma19143002)
Supplement: Supplementary file 1 [file materials-19-03002-s001.zip › materials-4391933-supplementary.pdf]

Supplementary Material for the manuscript

Data-Driven Prediction of Induced Voltage in CT-Based Magnetic Energy Harvesting Systems Considering Nonlinear B–H Characteristics

Seunggyun Byeon [1], Minjoong Kim [2] and Jihwan Song [1,*]

[1] Department of Mechanical Engineering, Sogang University, 35 Baekbeom-ro, Mapo-gu, Seoul 04107, Republic of Korea; sgbyeon@sogang.ac.kr
[2] Department of Mechanical Engineering, Hanbat National University, 125 Dongseodae-ro, Yuseong-gu, Daejeon 34158, Republic of Korea; kimminjoong.mmm@gmail.com
* Correspondence: jsong@sogang.ac.kr

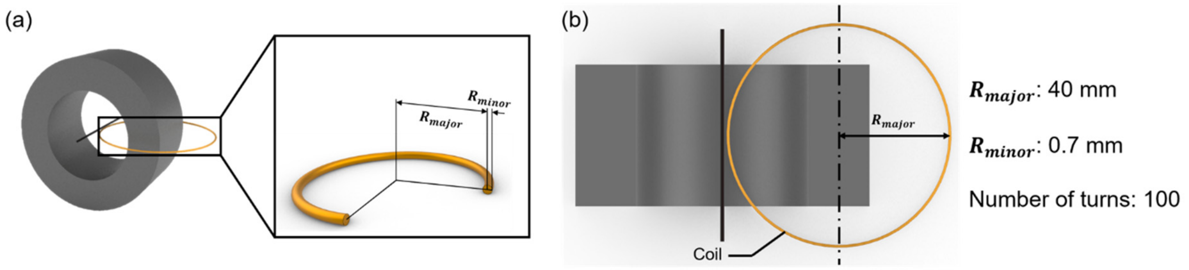

Figure S1. Coil geometry and placement in the CT-based MEH electromagnetic simulation model: **(a)** coil placement around the toroidal core and enlarged coil geometry; **(b)** schematic representation of the coil position relative to the core and power line, together with the coil geometric parameters and number of turns used in the simulation.

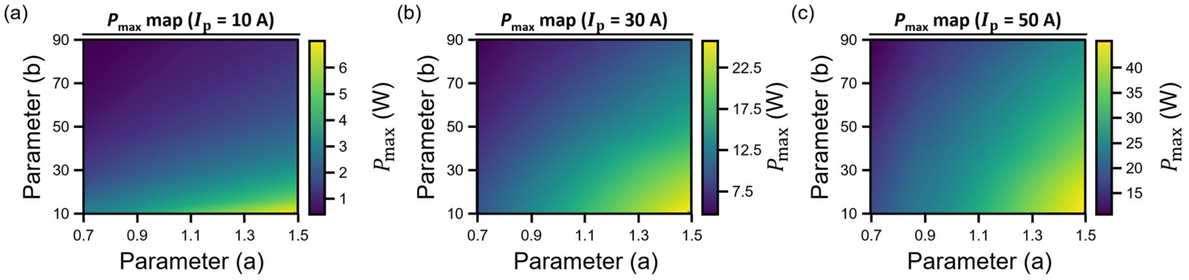

Figure S2. Comparison of P_{max} maps in the *a*–*b* parameter space under different primary-current conditions: **(a)** I_p = 10 A, **(b)** I_p = 30 A, and **(c)** I_p = 50 A, respectively.

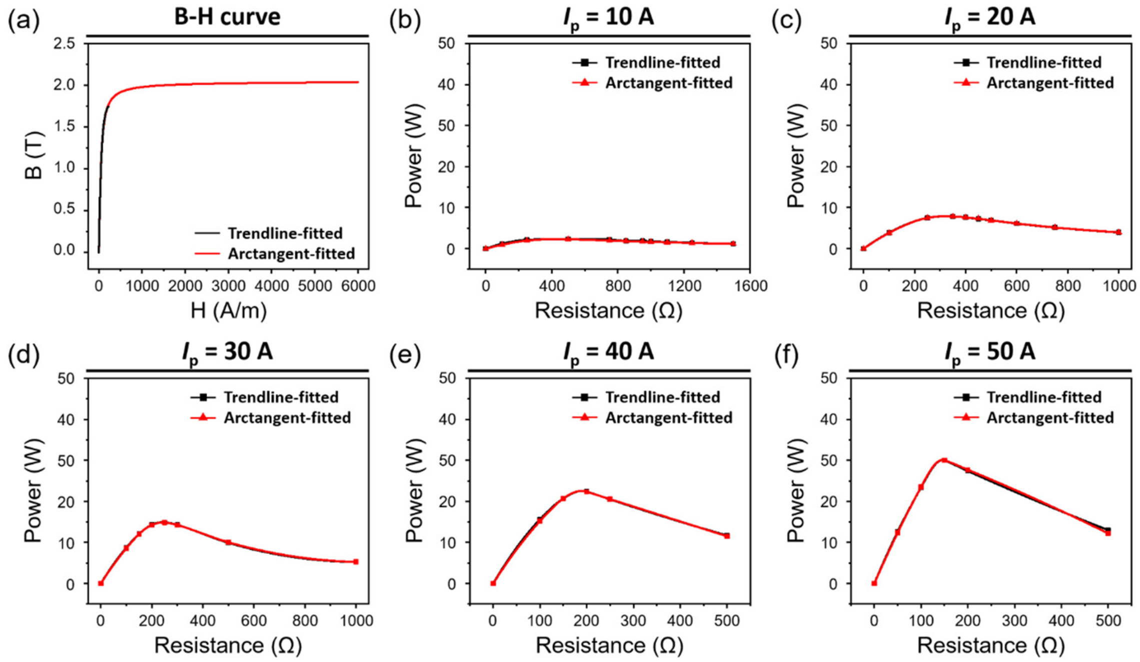

Figure S3. Arctangent-based B–H curve fitting and power–resistance comparison: **(a)** trendline-fitted and arctangent-fitted B–H curves; **(b–f)** simulated output power as a function of load resistance under primary-current conditions of **(b)** I_p = 10 A, **(c)** I_p = 20 A, **(d)** I_p = 30 A, **(e)** I_p = 40 A, and **(f)** I_p = 50 A.

Table S1. Error metrics of arctangent-based simulation results relative to experimental measurements.

MSE (W^2)	RMSE (W)	MAE (W)	R^2
6.816	2.611	1.823	0.809
